# Bibliometric Analysis of Granger Causality Studies

**DOI:** 10.3390/e25040632

**Published:** 2023-04-07

**Authors:** Weng Siew Lam, Weng Hoe Lam, Saiful Hafizah Jaaman, Pei Fun Lee

**Affiliations:** 1 Department of Physical and Mathematical Science, Faculty of Science, Kampar Campus, Universiti Tunku Abdul Rahman, Jalan Universiti, Bandar Barat, Kampar 31900, Perak, Malaysia; lamws@utar.edu.my (W.S.L.); pflee@utar.edu.my (P.F.L.); 2 Department of Mathematical Sciences, Faculty of Science and Technology, Universiti Kebangsaan Malaysia (UKM), Bangi 43600, Selangor, Malaysia

**Keywords:** Granger causality, bibliometric analysis, subject area, business economics, VOSviewer

## Abstract

Granger causality provides a framework that uses predictability to identify causation between time series variables. This is important to policymakers for effective policy management and recommendations. Granger causality is recognized as the primary advance on the causation problem. The objective of this paper is to conduct a bibliometric analysis of Granger causality publications indexed in the Web of Science database. Harzing’s Publish or Perish and VOSviewer were used for performance analysis and science mapping. The first paper indexed was published in 1981 and there has been an upward trend in the annual publication of Granger causality studies which are shifting towards the areas of environmental science, energy, and economics. Most of the publications are articles and proceeding papers under the areas of business economics, environmental science ecology, and neurosciences/neurology. China has the highest number of publications while the United States has the highest number of citations. England has the highest citation impact. This paper also constructed country co-authorship, co-analysis of cited references, cited sources, and cited authors, keyword co-occurrence, and keyword overlay visualization maps.

## 1. Introduction

Identifying causal network problems is important for effective policy and management, and recommendations on climate, epidemiology, and financial regulations. Identifying causality in complex systems can be difficult. Granger causality is an approach that uses predictability as opposed to correlation to identify causation between time series variables [1]. Variable X is said to “Granger cause” Y if the predictability of Y declines when X is removed from the universe of all possible causative variables. The key requirement of Granger causality is separability, namely that information about a causative factor is independently unique to that variable and can be removed by eliminating that variable from the model.

In statistics, Granger causality analyses the information flow between time series. Granger causality was proposed and named after the developer, Clive W. J. Granger in 1969 as a linear vector autoregressive (VAR) model in econometric time series analysis [1,2,3]. In Granger causality, a cause shall precede its effect while the understanding of a cause will help to increase the accuracy to predict the effect [4,5]. In a Granger causality test, a variable may be defined in two ways, namely Granger-cause and fail to Granger-cause. If a variable is identified as a Granger-cause, the variable brings down the forecasting error in the model. On the other hand, a variable will fail to Granger-cause other variables when its historical value cannot significantly predict the future value or when there is no statistical significance in the lagged values of the equation [6]. Therefore, a time series $\left\{x_{m,t}\right\}$ is a Granger-cause of another time series $\left\{x_{n,t}\right\}$ if the history of the time series $x_{m}$ enhances the prediction of $x_{n}$ over the time of $x_{n}$ alone. In year 2000, Schreiber [7] introduced the transfer entropy measure to quantify the statistical coherence between processes evolving over time.

With the availability of optimal estimation algorithms of VAR models, Granger causality has attracted the attention of researchers in several prominent research areas. VAR models also do not require any assumption on the physical mechanism for application. Moreover, permutation or bootstrapping are not needed to test the significance in Granger causality when the sample size is large. The result of a Granger causality test shall also remain consistent regardless of the overall signal strength [8,9,10]. Granger causality has been widely applied in several prominent areas. In neuroscience, Granger causality has been applied to functional magnetic resonance imaging (FMRI) [11,12,13], magnetoencephalography (MEG) [14,15,16], and local field potentials (LFP) [17,18,19]. Abdalbari et al. [20] used Granger causality for sleep data analysis to capture the physiological mechanisms during wakefulness and sleep. Hartmann et al. [21] studied the brain–heart interaction during sleep by using Granger causality to examine the electroencephalography (EEG) frequency bands with cortical and cardiovascular activities. Gao et al. [22] improved the classification of emotional states when Granger causality was applied with the Histogram of Oriented Gradient.

In finance, Granger causality has been used to determine the causal relationship between the financial development and economic growth in African countries [23]. The financial development and economic growth relationship has also been studied in the Middle East and North Africa (MENA) region [24]. Amano [25] studied the finance–development relationship during pre-war and post-war period in the United States, United Kingdom, and Japan. Fahimi et al. [26] investigated the globalization-driven energy consumption in Mexico, India, Nigeria, and Turkey. Candelon and Tokpavi [27] applied Granger causality in the European stock markets for spill over analysis. Coronado et al. [28] studied the cause–effect of COVID-19 news and stock market reactions in the United States and Latin America using Granger causality based on transfer entropy. Zuhud et al. [29] analysed the Malaysian stock market using Granger causality and transfer entropy. Granger causality has also been used to assess the dependency between different sectors with past time series [30]. Past studies have also used Granger causality to study Twitter sentiments’ effects on stock prices [31,32]. Granger causality has also been accepted in the areas of business, management, accounting, and economics [33,34,35,36,37,38,39,40,41]. The contribution of Granger causality has also been noticed in computer science [42,43,44,45,46] and engineering [47,48,49,50,51].

The popularity of Granger causality in these notable areas encourages further research and publications. To date, no bibliometric analysis has been done on Granger causality. Therefore, this paper aims to perform a bibliometric analysis on Granger causality using the Web of Science database. With the increasing availability of scientific databases and bibliometric software, bibliometric analysis has been used to assess scientific data and their research impact [52,53,54]. The research impact of the Granger causality publications obtained from the citation metrics, keywords, and authorships reflect the contribution of the publications in research and the industry. The research impacts, which are factual and objective, also show the practicality and popularity of a research area in current and future research.

This paper also examines the emerging trends in the papers, publication titles, research areas, and collaborations on Granger causality. From the emerging trends obtained from the bibliometric analysis, researchers may identify the hotspots of their research areas and determine the gaps for future works. The results of this bibliometric analysis of Granger causality are both objective and subjective. Performance analysis that shows the citation metrics of the documents and journals are objective findings while the scientific mapping that displays networks of relationships are subjective in nature [55,56]. This bibliometric analysis of Granger causality hopes to provide a comprehensive coverage on the publications of Granger causality and identify the research gaps to allow researchers to acquire ground-breaking ideas and position them to expand and enhance the application of Granger causality.

Section 2 continues with the literature review on the applications of Granger causality. Section 3 discusses the materials and methods used in the bibliometric analysis of Granger causality. Section 4 presents the results of the bibliometric analysis. Section 5 summarizes this paper with suggestions on future trends.

## 2. Literature Review on the Application of Granger Causality

Granger causality is applied in many areas such as business economics, environmental sciences, neurosciences, and computer sciences. This section shall review the literature on the application of Granger causality.

### 2.1. Business Economics

In business economics, Granger causality has been applied to perform a causality analysis on foreign direct investment (FDI), Islamic bank, and the capital market.

Zhang and Zhang [57] studied the impacts of gross domestic product, trade structure, exchange rates, and FDI inflow on carbon emissions in China. This paper found that FDI inflow positively affected the carbon emission rate in the country. Three policy implications were made in this paper. Firstly, the authors suggested that China should improve its high value-added low carbon-intensive areas such as the advertising, maintenance, and finance sectors. Secondly, the stability of the Chinese Yuan exchange rate could be promoted to lower carbon emissions as it will affect carbon demand and trigger price fluctuation. Thirdly, the government can direct FDI into low-carbon industries or high-tech sectors.

Jebli et al. [58] studied the causality among renewable energy usage, number of tourists, trade openness, economic growth, FDI, and carbon emissions in Central and South America. This paper concluded that tourism, renewable energy, and FDI reduced carbon emissions while trade and economic growth increased carbon emissions. Therefore, this paper proposed that policies such as FDI, usage of renewable energy, and developing green tourism could benefit the environment.

Granger causality was applied to identify the direction of causality to evaluate FDI, export, and economic growth in South Africa [59]. This paper confirmed that FDI and exports contributed to economic growth as there were unidirectional relationships from FDI to economic growth and FDI to exports. There was a bidirectional relationship between economic growth and export activities. The policy implications in this paper included the stimulation of FDI with investor incentives, developing a fair macroeconomic environment, and wise application of loose monetary policy in South Africa.

Arogundade et al. [60] analysed the human capital, institutional quality, FDI, and poverty in sub-Saharan Africa. This paper deduced that FDI had no direct relationship on the occurrence and level of poverty. FDI had negative effects on the absorptive capacity of the host country. This paper proposed several policies such as to invest in human capital and perform public sector reform to reduce corruption and improve political stability.

Farooqi and O’Brien [61] studied the effects of Basel standards on Islamic and conventional banks in the Gulf region. This paper concluded that the proposal of a market-based measure of bank stress led the accounting-based Tier-1 ratio and was in accordance to the Basel regulation’s Pillar 3. Therefore, the use of a measure to signal bank stress can be used to identify oncoming challenges before they escalate.

Setyowati [62] investigated the factors that affected saving and financing in Islamic banks in Indonesia. This paper found that there was a long-running cointegrating relationship in saving and financing to the consumer price index (CPI), manufacturing, interest rate, exchange rate, and Jakarta Islamic Index. Structural breaks occurred around January 2006 to April 2007 to signal financial crisis. There were bidirectional relationships between deposits and manufacturing, and between CPI and financing. Real activity, which was manufacturing, also affected Islamic bank financing. The policy implications in this paper were to provide incentives to encourage real activity such as production and manufacturing to allow Islamic banks to fulfil their intermediary roles and to implement an effective monetary policy on inflation to stabilize the market.

Samad [63] studied the relationship between Islamic banks’ return on depositors and conventional banks’ deposit interest in Bahrain. This paper found that there was a bidirectional relationship between Islamic banks’ returns on depositors and conventional banks’ interest rates.

Sharif et al. [64] assessed the time–frequency relationship between COVID-19 pandemic, oil prices, geopolitical risks, economic uncertainty, and the US stock market. This paper noted that the pandemic had a larger impact on the geopolitical risks and economic uncertainty compared to the US stock market. Oil was leading the US stock market regardless of the frequency. This paper suggested including a geopolitical risk index to analyse the financial impact of the pandemic. The US government should also design and implement a coherent economic strategy during and after the pandemic to foster market opening. Asset managers and investors should reassess the risk management framework to deal with the pandemic risks.

Wang et al. [65] studied the risk spill over effect from the US economic policy uncertainty (EPU) index, equity market uncertainty index, and Chicago Board Options Exchange’s CBOE Volatility Index (VIX) to Bitcoin. The authors found negligible risk spill over effects from these indices to Bitcoin. This study is beneficial to the investors when performing asset portfolio investment as Bitcoin diversifies extreme market shocks. This paper suggested that future studies could check if the cryptocurrency market is immune from EPU shocks.

Robiyanto et al. [66] measured the effectiveness of ASEAN-5 initiative from the portfolio perspective. The results showed weak integration into the equity markets in ASEAN-5. However, the ASEAN-5 initiative had an effect on the capital markets. This paper then suggested that investors in Malaysia, Singapore, and the Philippines should not invest excessively in Indonesia and Thailand equities when the market was unstable because Indonesia and Thailand were the net contributors to the volatility.

Athari and Bahreini [67] investigated the effects of economic policy uncertainty on travel and leisure companies’ debts in western Europe. Economic policy uncertainty had adverse effects on travel and leisure companies’ debt level. Countries with high economic policy uncertainty had lower debt ratios. The policy implications included the monitoring of the rise of economic policy uncertainty to stabilize cash flow for expansion and investment of travel and leisure companies, to perform portfolio diversification to reduce the adverse effects of economic policy uncertainty, and to consider firm and country-level matrices prior to setting debt ratios.

Athari [68] examined the causal relationship between financial inclusion and domestic political risk on the banking sector around the world. There were positive causal relationships from capital deregulation, credit risk inefficiency, market power, and indices in financial inclusion, political risk, and economic risk to banking stability. This paper suggested that countries should increase their stability by raising financial inclusion to offer stable environments for the economy and politics.

### 2.2. Environmental Sciences

In environmental sciences, Granger causality has been applied to assess ecological footprints, resource management, renewable energy, and carbon emissions.

Zafar et al. [69] investigated the relationship between natural resources, human capital, and FDI on the ecological footprint in the United States. Human capital and natural resources lowered the ecological footprint while economic growth and FDI elevated the ecological footprint. Several policy recommendations were given in this paper. Firstly, the government should monitor and control the excessive use of natural resources by encouraging its residents to reduce their consumption and adopt efficient products. The government should also encourage sustainable consumption and sustainable infrastructure for FDI. The United States should also attract high-tech FDI that does not make a large contribution to the ecological footprint. The country should also attract investments for renewable energy sources, infrastructure, and technology to improve bio-productivity.

Philip et al. [70] to study the best policies for Malaysia to achieve the 2030 climate goal to curb carbon emissions. This paper found that in the beginning of the economic growth, economic activities posed negative effects to the environment. However, the relationship was reversed at a later stage. Hence, this paper recommended the deregulation of the renewable energy sector to include private and public players. The government can also offer tax reductions and credits and encourage financial institutions to support the investment in renewable energy.

Xie et al. [71] studied the effects of mineral and forestry resource volatility on the economic performances around the world from 1985 to 2021. This paper found that mineral resources and forestry resources have a unidirectional causality to economic performances. However, the authors also argued that oversupply of natural resources is detrimental to the economy of a country. Renewable energy sources should be adequate to meet output objectives. Countries also need to practice efficient use of renewable energy resources to meet sustainability goals.

Adebayo [72] investigated the environmental consequences of fossil fuels in Spain. Fossil fuels lowered the environmental quality in the short and medium term, but renewable energy improved the quality of the environment. FDI enhanced the environmental quality, but economic complexity had detrimental effects on the quality of the environment. Based on outcome, the government can carry out schemes to attract FDI and multinational players to invest in green technologies. The government can also implement and tighten the environmental regulations for multinational companies currently operating in the country. Subsidies and tax reliefs for clean technologies can be introduced. The government should also encourage the shift to an energy mix to reduce reliance on fossil fuels and increase investment in renewable energy. Economic structural change can also be accelerated to improve the knowledge intensive sectors and production input mix for efficient performances.

Huang et al. [73] measured the causality between economic policies and carbon dioxide emissions in the European Union. This study aimed to find out the relationship between macroeconomic policies, national expenditure, non-renewable energy use, renewable energy use, and carbon dioxide emissions. Monetary tools had harmful effects on carbon emissions. The tightening of monetary policy could reduce the adverse effects of carbon emissions. Policy assessment also had a unidirectional relationship with energy use.

Nketiah et al. [74] studied the impacts of tourism, renewable energy, and biocapacity in enhancing or restricting the ecological footprints in West Africa. Human capital, natural resources, tourism, and real income had positive effects on the ecological footprints in West Africa. There was unidirectional causality from ecological footprints to renewable energy usage, human capital and urbanization. A bidirectional relationship was observed between biocapacity and real income. This paper suggested that countries in West Africa implement policies to deter increasing ecological footprints per capita and reduce overexploitation of natural resources.

### 2.3. Neurosciences

Granger causality has wide application in neurosciences such as cognitive, computational, and clinical neurosciences. Pesaran et al. [18] investigated large-scale brain dynamics. Granger causality was used because it was easy to apply to identify the direction of influence for temporal predictions in stochastic processes. Granger causality can also differentiate between direct and indirect influences. Wang et al. [75] studied the interacting brain networks when robot-assisted training and simulation were used. Granger causality was used to study the effective connectivity of the brain networks when while performing rehabilitation training tasks with a robotic device under various feedback conditions. Du et al. [76] aimed to find out whether a vibrotactile enhanced hand rehabilitation device could enhance sensorimotor brain activities. The training task of moving a hand and vibration simulation had strong causal influences between both sides of the cerebral hemispheres, and thus, had high training efficiency in functional cerebral hemodynamics.

Ye et al. [77] investigated the neural features of internet gaming disorder. Granger causality test revealed that there was connectivity from the right precentral gyrus to the left precentral gyrus and dorsal anterior cingulate cortex, which affected the internet gaming disorder severity. Zhang et al. [78] proposed the cross-frequency Granger causality feature extraction and fusion in both hemispheres for EEG emotion recognition. This proposed Granger causality had higher accuracy than the same-frequency band Granger causality features. Sysoev et al. [79] used Granger causality to describe the directed network activities between the somatosensory index and rostral reticular thalamic nucleus, caudal reticular thalamic nucleus, higher order thalamic nuclei and first order ventral posteromedial thalamic nucleus during sleep and wakefulness in rats. Ursino et al. [80] interpreted cortical signals reconstructed from EEG to study brain connectivity using temporal Granger causality in individuals with autism. Fu et al. [81] used neural Granger causality to examine the changes in schizophrenia’s non-linear causal couplings.

### 2.4. Computer Science

Lamsal et al. [82] used Granger causality to predict the COVID-19 daily cases in Australia from Twitter conversations. This study found that latent social media variables had extra prediction capability for forecasting models. Cai et al. [83] studied the causal effects of social bots on information diffusion in social networks for public health information in China. Granger causality found that sentiments of humans and social bots were able to predict each other. This paper then suggested that emergency managers shall control the bots at the end paths and act as opinion leaders to guide internet users’ sentiments. Tank et al. [84] used deep learning to propose non-linear dynamics of Granger causality with structured multilayer perceptrons or recurrent neural networks and sparsity inducing penalties on the weights.

Pirnay and Burnay [85] attempted to systemize the identification of causalities in Strategy Maps for risk management and decision-making in Belgium. A hybrid gray theory and Granger causality model was constructed for sensor correlation network structure mining on trains. After building the vehicle information network, the authors used complex network theory to mine the vehicle information network to find the causality between the nodes. Wang et al. [86] detected a causal structure for cloud services using Granger causality. Their results noted that neural Granger causality was better on linear and non-linear time series data. For greater linear time series, linear Granger causality was more efficient. Aviles-Cruz et al. [87] studied single user activities on smartphones with data obtained from accelerometer sensors. Zhang et al. [88] used Granger causality to identify the spatiotemporal causal relationship to obtain useful features for a deep learning multitask learning model for traffic speed prediction.

Therefore, with the broad application of Granger causality and its policy implications in business, society, and public administration, this paper intends to perform a bibliometric analysis of Granger causality.

## 3. Materials and Methods

**Figure 1:**
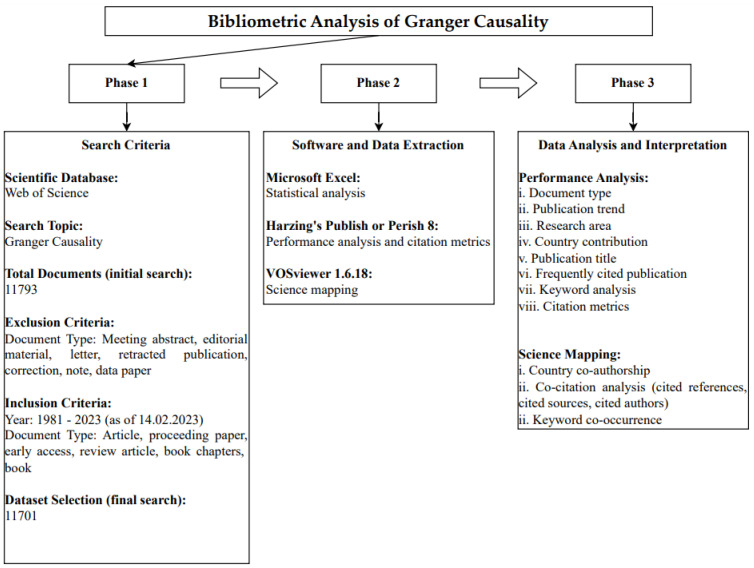
Bibliometric analysis process flow.

The objective of this paper is to perform a bibliometric analysis of Granger causality. Web of Science, which is a credible and globally accepted scientific database for peer-reviewed publications, was used to extract the publication data of Granger causality [89,90,91,92]. The process of this bibliometric analysis can be explained in three steps as shown in Figure 1 [93].

In the beginning, “Granger causality” was selected for this bibliometric analysis. Then, a search based on the following query: (“Granger causality” (Topic)) was made on the Web of Science database to extract the publication data on Granger causality on 14 February 2023. A total of 11,793 documents appeared from this query. After that, a filter was applied to identify articles, proceeding papers, early access, review articles, book chapters, and books only. The final dataset which was extracted in plain text file format consisted of 11,701 publications from 1981 to 2023.

Then, performance analysis and scientific mapping were performed. Performance analysis states the contributions of documents and journals, which are quantifiable. Important measures in performance analysis include total number of publications (TP), total number of citations (TC), number of cited papers (NCP), average number of citations per paper (C/P), average number of citations per cited paper (C/CP), *h*-index, and *g*-index. The details on the publication number indicates productivity level while the citation metric is a measurement of impact. The value of *h*-index is defined as the *h* number of publications with at least *h* citations. The value of *g*-index is explained as the *g* number of publications with at least *g^2^* citations. Thus, the *h*-index measures the influence while *g*-index measures the impact [94,95,96,97].

Scientific mapping describes the relationships, connections, and interactions in research. The frequently used scientific mappings are country co-authorship and keyword co-occurrence maps [98]. A co-authorship map studies the scientific interaction among researchers in a topic. Co-authorships improve research contribution as experts from various fields collaborate to share knowledge, funding, and technology. Co-citation analysis shows the references, sources, and authors which are commonly cited together in other documents. A keyword co-occurrence map shows the thematic relationships of the keywords used and could be used to determine the current research trends. The combination of performance analysis and scientific mapping enriches the results of bibliometric analysis. In this paper, the performance analysis was performed with Harzing’s Publish or Perish 8 while the scientific mapping was done with VOSviewer [99,100,101,102].

## 4. Results

This section presents the results of the bibliometric analysis of Granger causality. Table 1 explains the document types of the Granger causality publications. A total of 78.52% of the publications are articles and 16.30% of the publications are proceeding papers. Early access, review articles, book chapters, and books make up 2.41%, 1.96%, 0.78%, and 0.03% of the total publications, respectively.

### 4.1. Publication Trend

Table 2 shows the publication trend of Granger causality documents from 1981 to 2023 as of 14 February 2023. The first paper indexed in the Web of Science database is titled “Granger-causality in multiple time series” by TjØstheim [103]. This paper, which was published in 1981, has received 21 citations to date. This paper studied the problem of constrained estimation in models with a known causality structure. The second paper listed on the Web of Science database is “Granger causality and the time series analysis of political relationships” by Freeman [104], which was published in 1983 with 168 citations as of 14 February 2023. This paper discussed the application of Granger causality in the study of political relationships. The third indexed paper is “Granger causality and policy effectiveness” authored by Buiter [105] in 1984, which has received 18 citations. This paper discussed the Granger causality and policy effectiveness with an optimizing controller as well as the Granger causality and automatic stabilizers. Another paper titled “A note on tests of Granger causality” was also published in 1984. This paper by Bessler and Kling [106] has received 18 citations. This paper studied the issue of the relationship between the annual economic activity and annual sunspots.

**Figure 2:**
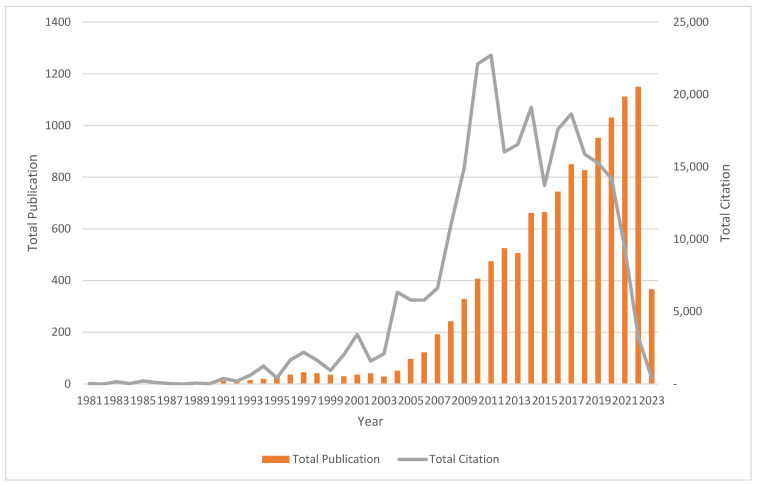
Publication and citation trends of Granger causality.

The annual number of publications from 1981 to 1990 were low at only a single digit each year. There was a small fluctuation in the number of publications from 1991 to 2005. However, after 2005, there was a spike in the overall annual publication rate until 2022. The highest number of papers was also recorded in 2022 with 1150 documents. Therefore, it is predicted that the number of documents published in 2023 will exceed the number of documents in 2022, as Granger causality received notable attention from researchers. Table 2 tabulates the annual publication and citation metrics of Granger causality documents from 1981 to 2023. The publication and citation trend of Granger causality publications are described in Figure 2.

From Table 2, the highest total number of citations (TC) was recorded in 2011 with 22,709 citations. There are four papers among the top 10 cited papers which were published in 2011 that contributed to the high number of total citations. The paper by Friston [107] titled “Functional and effective connectivity: a review” was the third most cited paper with 1862 citations. The fifth most cited paper titled “Twitter mood predicts the stock market” by Bollen et al. [108] has received 1225 citations. The sixth most cited paper is “Network modelling methods for fMRI” by Smith et al. [109] with 1209 citations. The paper “FIAR: an R package for analyzing functional integration in the brain” by “Roelstraete and Rosseel [13] received 1055 citations.

The highest number of citations per paper (C/P) and highest number of citations per cited paper (C/CP) were achieved by the only paper “Granger causality and the time series analysis of political relationships” by Freeman [104] published in 1983. This paper has received 168 citations. The *h*-index was the highest at 70 in 2010, 2011, and 2014. This means that 70 papers have been cited at least 70 times in 2010, 2011, and 2014. The highest *g*-index of 144 was recorded in 2010 and 2011. There were 144 documents with a total of 20,736 citations published in 2010 and 2011.

### 4.2. Research Area

The 11701 publications have been classified into several research areas. Most of the publications were under business economics (5119 documents), environmental science and ecology (1300 documents), neurosciences/neurology (1133 documents), engineering (1122 documents), computer science (901 documents), science technology other topics (849 documents), energy fuels (745 documents), mathematics (560 documents), social science other topics (479 documents), and physics (343 documents). The top 10 research areas are listed in Table 3.

The Web of Science database has also classified the publications into several categories. The Web of Science database identified the top 10 categories of publications in the following order: economics (3717 documents), business finance (1109 documents), neurosciences (1036 documents), environmental sciences (948 documents), business (747 documents), energy fuels (745 documents), management (736 documents), environmental studies (599 documents), green sustainable science technology (499 documents), and engineering—electrical/electronic (457 documents). The top 20 Web of Science categories are shown in Table 4.

### 4.3. Country Contribution

Researchers from about 150 countries have contributed to the publication of Granger causality from 1981 to 2023. The top 3 countries with the highest total publication (TP) were China (3114 documents), the United States (2113 documents), and Turkey (874 documents). Among the 274,321 total citations received from the 11701 documents, the United States received the highest number of citations with 80055 citations, which was more than 29% of the total citations. Meanwhile, England had the most impactful publications with 46.64 citations per paper (C/P) and 51.66 citations per cited paper (C/CP). The United States had the highest *h*-index (125) and *g*-index (225). A total of 125 documents have been cited more than 125 times while 225 documents have a cumulative number of more than 50,625 citations. Even though China had the highest total number of publications (TP), the publication impact (measured by C/P and C/CP) of these publications fell behind England, Malaysia, the United States, Turkey, Pakistan, Australia, Germany, and Italy. Table 5 demonstrates the top 10 countries with the high contributions in Granger causality publications.

The country co-authorship diagram generated by VOSviewer shows the synergy of research collaboration among countries. The node size in the country co-authorship diagram reflects the strength of co-authorship. That is, when a country has high co-authorship with other countries, the node of the country will be large [110]. The colour of the node and line show the country cluster [111]. The thickness of the line connecting the two nodes indicates the co-authorship link strength. The link strength is the number of publications with co-authorship between two countries. A shorter line also indicates a stronger link [112]. The total link strength of a country reflects the co-authorship strength of the country with all other countries [113,114].

Table 6 explains the top 10 countries with the highest number of co-authorships. The United States had the highest total link strength of 1454 documents from their total of 2113 publications, followed by China (1386), England (840), Pakistan (686), France (617), Turkey (572), Germany (515), Australia (487), Malaysia (444), and Italy (409). This means that the United States had the highest number of collaboration with other countries.

**Figure 3:**
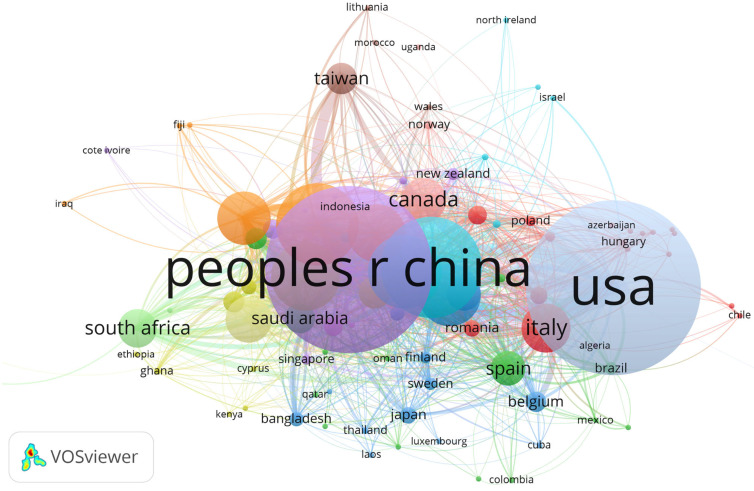
Country co-authorship analysis of Granger causality publications.

Figure 3 depicts the country co-authorship diagram of Granger causality publications. The United States had the largest node followed by China because these two countries have high total link strengths with 1454 and 1386 documents, respectively. The largest link strength of 292 was between the United States and China. This means that the United States and China had the highest number of co-authorships between countries and the line between them is the thickest. China and Pakistan had the second highest link strength of 188, followed by the United States and England (102), the United States and Germany (100), China and England (93), and China and Taiwan (86).

There were 13 clusters in total. The first cluster is in bright red and made up of Argentina, Azerbaijan, Bosnia and Herzegovina, Chile, Croatia, Czech Republic, Greece, Hungary, Italy, Macedonia, Poland, Romania, Serbia, Slovakia, Slovenia, Switzerland, and Ukraine. The second cluster is dark green and consists of Bahrain, Brazil, Columbia, Ecuador, Egypt, Jordan, Mexico, Oman, Portugal, Qatar, Saudi Arabia, Spain, Tunisia, and Uruguay. The third cluster is dark blue and contains Bangladesh, Belgium, Cuba, Finland, Germany, Japan, Laos, Luxembourg, Netherlands, Scotland, Sweden, Thailand, and Uzbekistan. The fourth cluster is a dark yellow with Cameroon, Cyprus, Ethiopia, Ghana, Kenya, Nigeria, Russia, Turkey, and Vietnam. The fifth cluster is dark purple and made up of Côte d’Ivoire, Indonesia, Iran, Kazakhstan, New Zealand, China, Philippines, Singapore, and South Korea. The sixth cluster is in bright blue with Denmark, England, France, Ireland, Israel, Lebanon, North Ireland, and Sri Lanka. The seventh cluster (orange) consists of Australia, Bulgaria, Fiji, Iraq, Malaysia, and Pakistan. The brown cluster contains Latvia, Lithuania, Morocco, Taiwan, and Wales. Algeria, Austria, Kuwait, and the United Arab Emirates make up the light purple-pink cluster. Canada, Norway, and Uganda are in the light red cluster. Brunei, South Africa, and Zimbabwe are in the light green cluster. The United States and Barbados are in one cluster. India and Peru make up one cluster.

### 4.4. Journals

There are about 200 journals that have published documents on Granger causality. Table 7 displays the top 10 journals of Granger causality publications. Clarivate Analytics assesses the importance of a journal by measuring the frequency an average document in a journal is cited in a year with the use of Journal Impact Factor (JIF). Journals in the Science Citation Index Expanded (SCIE) and Social Sciences Citation Index (SSCI) have JIFs. JIFs can be used to compare journals within the same field. A JIF is defined as the average number of citations obtained per document published in the journal in the past two years [115]. For example, the JIF 2021 for the journal *Renewable & Sustainable Energy Reviews* was 16.799. This means that the average number of citations received in 2021 for the documents published in this journal in 2019 and 2020 was 16.799 [116].

The Journal Citation Index (JCI), which is also computed by Clarivate Analytics, offers a single journal level index to compare journals across all fields. All the journals in the Web of Science database have a JCI even though some of the journals do not have a JIF [117]. For JCI 2021, the analysed years are 2018, 2019, and 2020. The global baseline value is 1.00. Therefore, a JCI higher than 1.00 indicates a higher than average citation impact while a JCI lower than 1.00 indicates a lower than average citation impact. For example, *Energy Economics* received a JCI 2021 of 3.05. This means that, across this journal, the published documents received 3.05 times more citations than the average in the field [118].

CiteScore is a measurement of the journal impact factor by the Scopus database. CiteScore includes document types such as articles, conference papers, book chapters, and data papers. CiteScore is calculated by using the number of citations obtained by a journal in a year to the number of documents published in the last four years, divided by the total document listed in the Scopus database during those four years [119]. For example, *Renewable & Sustainable Energy Reviews* has a CiteScore 2021 of 28.5. This means that, the average number of citations obtained in 2021 for the documents published in 2017, 2018, 2019, and 2020 was 28.5 [120].

The SCImago Journal Rank (SJR) evaluates journals indexed in the Scopus database by assigning weights to the citations with regard to the journal’s importance. Therefore, citations in highly impactful journals receive greater weights that citations in less important journals. SJR is the average number of weighted citations obtained during a year per paper published in the past three years. For example, the SJR 2021 for *Renewable & Sustainable Energy Reviews* was 3.678. Documents published in 2018, 2019, and 2020 in this journal obtained 3.678 weighted citations during 2021. This value also shows that *Renewable & Sustainable Energy Reviews* has high prestige [121].

The Source Normalized Impact per Paper (SNIP) examines the citation impact by calculating the weighted citations with regard to the total citations in a field. SNIP considers field-specific variation in citations. In a field where a citation is less probable, the impact of the citation will be assigned a greater weight. Likewise, where the citation potential is high in the field, the impact of the citation will be lower [122,123]. Based on Table 7, *Environmental Science and Pollution Research* (260) published the most documents on Granger causality, followed by *Energy Economics* (174), *Applied Economics* (156), *Neuroimage* (154), *Sustainability* (150), *PLoS One* (118), *Applied Economics Letters* (113), *Economic Modelling* (111), *Resources Policy* (107), and *Renewable & Sustainable Energy Reviews* (103).

### 4.5. Most Cited Publication

Table 8 reveals the top 10 most cited publications on Granger causality. The most cited document “Saliency, switching, attention and control: a network model of insula function” by Menon and Uddin [92] received 2603 citations. This paper presented a network model that the insula is sensitive to salient events and its core function is to mark such events for extra processing and initiate appropriate control signals. The second most cited document “Elements of Causal Inference: Foundations and Learning Algorithms” by Peters et al. [93] studied the concept of causality and related problems. The third most cited paper by Friston [75] reviewed the development and practice of connectivity analyses in neuroimaging, functional connectivity, causal modelling, connectomics, and multivariate analyses of distributed patterns of brain responses. Sridharan et al. [94] used functional magnetic resonance imaging to study the mechanisms underlying the switching of brain networks in experiments. The next most cited paper by Bollen et al. [76] applied a Granger causality analysis and fuzzy neural network to investigate the hypothesis that public mood states are predictive of changes in Dow Jones Industrial Average (DJIA) closing values. The result showed that the accuracy of DJIA predictions can be significantly improved by the inclusion of specific public mood dimensions.

The sixth most cited paper by Smith et al. [77] studied the comparison among connectivity estimation approaches by using simulated FMRI data. The seventh most cited paper by Baccala and Sameshima [95] presented a new frequency-domain approach based on the concept of Granger causality to describe the relationships between multivariate time series based on the decomposition of multivariate partial coherences determined from multivariate autoregressive models. The next most cited paper by Nolte et al. [96] presented an approach that is insensitive to false connectivity arising from volume conduction for interpreting EEG data in terms of brain connectivity. Roelstraete and Rosseel [13] introduced a functional integration analysis in an R (FIAR) package to perform various techniques for studying integration in brain networks. Sugihara et al. [97] proposed a method based on nonlinear state space reconstruction which can distinguish causality from correlation.

**Figure 4:**
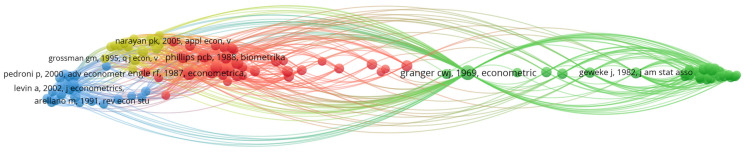
Co-citation analysis of cited documents on Granger causality.

The co-citation analysis of cited references shows the prestigious documents which are frequently cited together from another document [130]. Figure 4 shows the co-citation analysis of cited references on Granger causality. The papers by Granger [1] and Engle and Granger [131] were cited 683 times in the 11701 documents on Granger causality. These two papers also have the highest link strengths of 16100 and 11346, respectively. The papers by Engle and Granger [131] and Johansen and Juselius [132] also have a high co-citation with a link strength of 675. The papers by Phillips and Perron [133] and Dickey and Fuller [134] also have high co-citation with a link strength of 652. Another set of papers with high co-citation and link strength of 587 are by Granger [1] and Dickey and Fuller [134]. The papers by Engle and Granger [131] and Dickey and Fuller [134] were also highly co-cited together with a link strength of 571. The papers by Granger [1] and Geweke [8] were also frequently cited together in other documents with a link strength of 507. The papers by Phillips and Perron [133], Dickey and Fuller [134], and Johansen and Juselius [132] also have high link strengths of 7666, 7488, and 6726, respectively.

**Figure 5:**
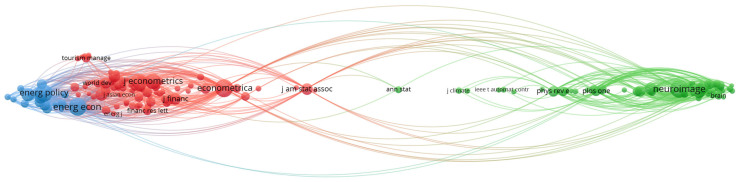
Co-citation analysis of cited source on Grange causality.

The co-citation of cited sources was also performed using VOSviewer and is shown in Figure 5. *Energy Economics* has the highest total link strength of 545250, followed by *NeuroImage* (517050), and *Energy Policy* (505603). *Econometrica* (332271), *Renewable & Sustainable Energy Reviews* (294434), *Journal of Econometrics* (274903), *Journal of Neuroscience* (207006), *Proceedings of the National Academy of Sciences* (202995), *Environmental Science and Pollution Research* (199643), and *Energy* (192916) also have high total link strengths.

**Figure 6:**
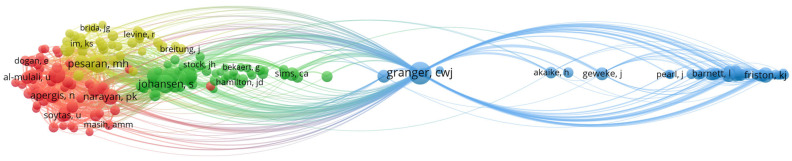
Co-citation analysis of cited authors in Granger causality publications.

The co-citation analysis of cited authors shows the main contributors in the field of Granger causality [130]. The top 10 co-cited researchers are Granger, C.W.J. (6762 citations, 61075 total link strength), Shahbaz, M. (2369 citations, 54951 total link strength), Pesaran, M.H. (3394 citations, 53148 total link strength), Apergis, N. (1867 citations, 46640 total link strength), Narayan, P.K. (2046 citations, 42402 total link strength), Johansen, S. (3296 citations, 41108 total link strength), Engle, R.F. (2548 citations, 31723 total link strength), Phillips, P.C.B. (1888 citations, 28485 total link strength), Ozturk, I. (1019 citations, 27371 total link strength), and Dickey, D.A. (2175 citations, 26510 total link strength). Figure 6 portrays the co-citation analysis of the cited authors in Granger causality publications.

### 4.6. Keyword Analysis

**Figure 7:**
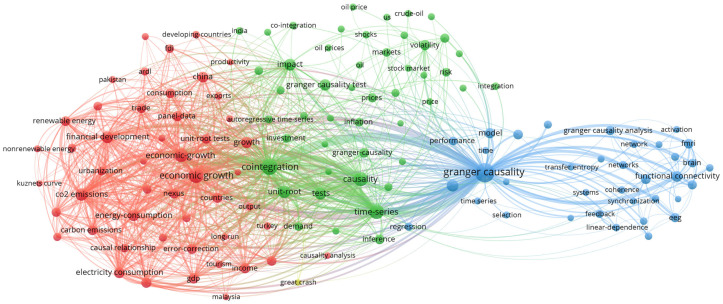
Keyword co-occurrence map of Granger causality publications.

VOSviewer also provides the keyword co-occurrence map to study the relationships between the keywords to allow scholars to determine the key ideas and the linkage of these key ideas in creating a sub-field for current and future research trends [135,136]. Table 9 shows the top 10 keywords with the respective total link strengths in Granger causality. “Granger causality” occurred 3963 times in all the 11,701 documents with a total link strength of 12,891, followed by “cointegration” (2128 occurrences, 11,166 total link strength) and economic growth (1590 occurrences, 8321 total link strength). Figure 7 depicts the keyword co-occurrence map of the Granger causality publications.

Based on Figure 7, the keywords can be classified into four clusters. The first cluster is red and consists of autoregressive distributed lag (ARDL), carbon emissions, causal relationship, causality analysis, China, economic growth, energy consumption, environmental Kuznets curve (EKC), error correction, exports, foreign direct investment (FDI), financial development, gross domestic product (GDP), globalization, hypothesis, income, Malaysia, non-renewable energy, OECD countries, panel cointegration, panel data, tourism, trade, Turkey, unit root tests, and urbanization. The second cluster, which is green, is made up of autoregressive time series, COVID-19, crude oil, dependence, determinants, efficiency, energy, exchange rate, granger causality, impact, India, inference, inflation, investment, market, monetary policy, oil price, return, risk, stock market, stock returns, transmission, unemployment, United States, vector autoregressive (VAR) model, vector error correction model (VECM), vector autoregressions, and volatility. The third cluster is blue and contains activation, brain, coherence, cortex, dynamics, EEG, effective connectivity, feedback, fMRI, functional connectivity, information flow, oscillations, prefrontal cortex, synchronization, and transfer entropy. The final cluster is in yellow and consists of great crash.

**Figure 8:**
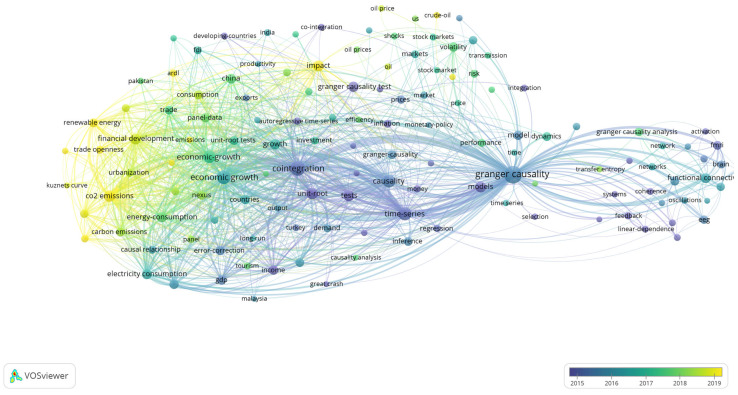
Keyword overlay visualization map of Granger causality publications.

The keyword overlay visualization map in Figure 8 explains the trend of Granger causality publications over time. The colour of the node and line represent the year of the documents contained the keywords were published. A lighter colour of the node and line show the recent focus of keywords in Granger causality [137]. The recent research in Granger causality focused on renewable energy, non-renewable energy, trade openness, carbon emissions, ARDL, EKC, and globalization. This shows that Granger causality is increasingly being applied to study environmental science, energy, and economics. The current trend in Granger causality also shows that many studies are involved in two or more research areas to broaden the application of Granger causality. Moreover, many current studies have focused on green and sustainability in their papers, such as in business, energy, and transportation. For example, nuclear energy and renewable energy have been studied to assess their impacts on the ecological quality in the United States [138], economic sustainability has been studied together with green resources, digitization, and financial development in Vietnam [139] while energy consumption has also been investigated together with tourist arrivals in the Middle East [140].

### 4.7. Citation Metrics

Citation metrics of the 11,701 documents of Granger causality publications from 1981 to 2023 as of 14 February 2023 have been extracted from Harzing’s Publish or Perish software and presented in Table 10. A total of 274,321 citations were received from the 11,701 documents with an *h*-index of 209 and *g*-index of 330.

## 5. Conclusions

The objective of this paper was to conduct a bibliometric analysis of Granger causality publications indexed in the Web of Science database from 1981 to 2023 as of 14 February 2023. The bibliometric analysis of Granger causality provided a comprehensive overview of the publication trends, research impact, and emerging trends in the various research areas. The first paper indexed in 1981 is titled “Granger-causality in multiple time series” by TjØstheim [103]. The highest number of publications was in 2022 with 1150 documents. The highest total citation was recorded in 2011 with 22709 citations. The most frequently cited paper was “Saliency, switching, attention and control: a network model of insula function” by Menon and Uddin [124] which was published in 2010 and has received 2603 total citations to date.

Most of the publications are articles and proceeding papers. The application of Granger causality can be found mostly in the top three subject areas which are business economics, environmental science and ecology, and neurosciences/neurology. China had the highest number of publication and number of cited papers. However, China’s publication impact was low compared to England, Malaysia, the United States, Turkey, Pakistan, Australia, Germany, and Italy. The United States had the highest total number of citations while England has the highest number of citations per paper and citations per cited paper. The top journal that published Granger causality documents was *Environmental Science and Pollution Research* by Springer Heidelberg with 260 total publication, 7631 total citations, and a JIF 2021 of 5.190.

The country co-authorship map found that the United States and China had the highest international coloration among researchers in Granger causality publications. The paper “Investigating Causal Relations by Econometric Models and Cross-Spectral Methods” by Granger [1] was the highest co-cited reference. The highest co-cited source was *Energy Economics*. The top three co-cited researchers were Granger, C.W.J., Shahbaz, M., and Pesaran, M.H. The most used keyword was “Granger causality”. The current focus of Granger causality research is on environmental science, energy, and economics. This study has a few limitations. Firstly, this study mainly focuses on the Web of Science database. Therefore, future studies may consider other databases for a bibliometric analysis of Granger causality. Secondly, the Web of Science database is continuously updating the new publications from time to time. Hence, a bibliometric analysis of Granger causality may be revisited in the future.

## Figures and Tables

**Table 1:** Document types of Granger causality publications.

Document Type	Total Publication (TP)	Percentage (%)
Article	9582	78.52
Proceeding Paper	1989	16.30
Early Access	294	2.41
Review Article	239	1.96
Book Chapters	95	0.78
Book	4	0.03

**Table 2:** Annual publication and citation metrics of Granger causality publication.

Year	TP	Percentage (%)	Cumulative Percentage (%)	NCP	TC	C/P	C/CP	*h*-Index	*g*-Index
1981	1	0.01	0.01	1	21	21.00	21.00	1	1
1983	1	0.01	0.02	1	168	168.00	168.00	1	1
1984	2	0.02	0.03	2	36	18.00	18.00	2	2
1985	2	0.02	0.05	2	211	105.50	105.50	2	2
1986	2	0.02	0.07	2	103	51.50	51.50	1	2
1987	3	0.03	0.09	2	33	11.00	16.50	2	3
1988	1	0.01	0.10	1	2	2.00	2.00	1	1
1989	3	0.03	0.13	3	63	21.00	21.00	2	3
1990	2	0.02	0.15	2	21	10.50	10.50	2	2
1991	12	0.10	0.25	11	386	32.17	35.09	6	12
1992	14	0.12	0.37	12	202	14.43	16.83	5	14
1993	15	0.13	0.50	15	603	40.20	40.20	9	15
1994	20	0.17	0.67	18	1238	61.90	68.78	11	20
1995	25	0.21	0.88	24	418	16.72	17.42	11	20
1996	37	0.32	1.20	36	1657	44.78	46.03	17	37
1997	45	0.38	1.58	42	2180	48.44	51.90	16	45
1998	42	0.36	1.94	38	1633	38.88	42.97	21	40
1999	36	0.31	2.25	32	948	26.33	29.63	13	30
2000	30	0.26	2.50	28	2015	67.17	71.96	15	30
2001	36	0.31	2.81	35	3428	95.22	97.94	22	36
2002	42	0.36	3.17	40	1576	37.52	39.40	21	39
2003	29	0.25	3.42	23	2077	71.62	90.30	16	29
2004	52	0.44	3.86	45	6332	121.77	140.71	28	52
2005	97	0.83	4.69	85	5795	59.74	68.18	35	76
2006	122	1.04	5.73	95	5789	47.45	60.94	34	75
2007	191	1.63	7.37	131	6626	34.69	50.58	41	80
2008	242	2.07	9.44	171	10,975	45.19	63.95	52	103
2009	328	2.80	12.24	225	14,951	45.58	66.45	60	118
2010	407	3.48	15.72	298	22,117	54.34	74.22	70	144
2011	474	4.05	19.77	374	22,709	47.91	60.72	70	144
2012	525	4.49	24.25	418	16,035	30.54	38.36	60	112
2013	506	4.32	28.58	433	16,551	32.71	38.22	65	112
2014	661	5.65	34.23	536	19,111	28.91	35.65	70	117
2015	664	5.67	39.90	545	13,713	20.65	25.16	59	95
2016	744	6.36	46.26	619	17,606	23.66	28.44	62	104
2017	850	7.26	53.53	690	18,654	21.95	27.03	63	111
2018	827	7.07	60.59	708	15,866	19.19	22.41	60	94
2019	952	8.14	68.73	811	15,259	16.03	18.82	56	90
2020	1031	8.81	77.54	878	14,196	13.77	16.17	54	85
2021	1112	9.50	87.04	882	9384	8.44	10.64	42	61
2022	1150	9.83	96.87	635	3221	2.80	5.07	23	32
2023	366	3.13	100.00	125	452	1.23	3.62	10	13
Total	11701	100			274,361				

**Table 3:** Research areas of Granger causality publications.

Research Areas	Total Publication
Business Economics	5119
Environmental Science and Ecology	1300
Neurosciences/Neurology	1133
Engineering	1112
Computer Science	901
Science Technology, Other Topics	849
Energy Fuels	745
Mathematics	560
Social Science, Other Topics	479
Physics	343
Radiology Nuclear Medicine and Medical Imaging	309
Mathematical Methods in Social Sciences	281
Operations Research Management Science	264
Public Administration	224
Psychology	197
Mathematical Computational Biology	140
Agriculture	135
Biochemistry and Molecular Biology	124
Automation Control Systems	117
Thermodynamics	107

**Table 4:** Web of Science categories of Granger causality publications.

Web of Science Categories	Total Publication
Economics	3717
Business Finance	1109
Neurosciences	1036
Environmental Sciences	948
Business	747
Energy Fuels	745
Management	736
Environmental Studies	599
Green Sustainable Science Technology	499
Engineering—Electrical/Electronic	457
Computer Science Artificial Intelligence	349
Multidisciplinary Sciences	347
Engineering—Biomedical	311
Radiology Nuclear Medicine and Medical Imaging	309
Computer Science Information Systems	296
Computer Science Interdisciplinary Applications	296
Social Science Mathematical Methods	281
Neuroimaging	274
Statistics Probability	268
Operations Research Management Science	264

**Table 5:** Top 10 countries with high contributions in Granger causality publications.

Country	TP	NCP	TC	C/P	C/CP	*h*-Index	*g*-Index
China	3114	2079	46,778	15.02	22.5	97	147
United States	2113	1876	80,055	37.89	42.67	125	225
Turkey	874	704	31,373	35.9	44.56	88	157
India	703	517	10,049	14.29	19.44	46	82
England	648	585	30,221	46.64	51.66	83	159
Pakistan	496	433	17,202	34.75	39.73	66	118
Australia	490	437	17,024	34.74	38.96	63	117
Italy	476	423	12,660	26.6	29.93	58	96
Germany	469	412	12,826	27.35	31.13	57	96
Malaysia	454	379	18,733	41.26	49.43	71	128

**Table 6:** Top 10 countries with co-authorships with other countries.

Country	Total Publication	Total Link Strength
United States	2113	1454
China	3114	1386
England	648	840
Pakistan	496	686
France	383	617
Turkey	874	572
Germany	469	515
Australia	490	487
Malaysia	454	444
Italy	476	409

**Table 7:** Journals of Granger causality documents.

Publication Title	TP	%	TC	Publisher	JIF	JCI	Cite Score	SJR	SNIP	*h*-Index
*Environmental Science and Pollution Research*	260	2.22	7631	Springer Heidelberg	5.190	0.81	6.6	0.831	1.154	132
*Energy Economics*	174	1.49	18,514	Elsevier	9.252	3.05	11.3	2.549	2.347	168
*Applied Economics*	156	1.33	3028	Routledge Journals, Taylor & Francis Ltd.	1.916	0.63	2.8	0.563	1.086	91
*Neuroimage*	154	1.32	10,816	Academic Press Inc. Elsevier Science	7.400	1.64	11.2	2.746	2.099	381
*Sustainability*	150	1.28	1673	Multidisciplinary Digital Publishing Institute	3.889	0.65	5.0	0.664	1.310	109
*PLoS One*	118	1.01	2993	Public Library Science	3.752	0.88	5.6	0.852	1.368	367
*Applied Economics Letters*	113	0.97	1481	Routledge Journals, Taylor & Francis Ltd.	1.287	0.41	1.8	0.4	0.663	54
*Economic Modelling*	111	0.95	6072	Elsevier	3.875	1.28	4.8	1.065	1.733	87
*Resources Policy*	107	0.91	2832	Elsevier Sci Ltd.	8.222	1.63	7.6	1.461	1.996	80
*Renewable & Sustainable Energy Reviews*	103	0.88	12,603	Pergamon-Elsevier Science Ltd.	16.799	1.26	28.5	3.678	4.535	337

**Table 8:** Top 10 most cited publications on Granger causality.

Title	Year	TC	Journal Title
Saliency, switching, attention and control: a network model of insula function [124]	2010	2603	*Brain Structure & Function*
Elements of Causal Inference: Foundations and Learning Algorithms [125]	2017	2131	*Elements of Causal Inference: Foundations and Learning Algorithms*
Functional and Effective Connectivity: A Review [107]	2011	1862	*Brain Connectivity*
A critical role for the right fronto-insular cortex in switching between central-executive and default-mode networks [126]	2008	1841	*Proceedings of the National Academy of Sciences of the United States of America*
Twitter mood predicts the stock market [108]	2011	1225	*Journal of Computational Science*
Network modelling methods for FMRI [109]	2011	1209	*NeuroImage*
Partial directed coherence: a new concept in neural structure determination [127]	2001	1168	*Biological Cybernetics*
Identifying true brain interaction from EEG data using the imaginary part of coherency [128]	2004	1060	*Clinical Neurophysiology*
FIAR: An R Package for Analyzing Functional Integration in the Brain [13]	2011	1055	*Journal of Statistical Software*
Detecting Causality in Complex Ecosystems [129]	2012	1046	*Science*

**Table 9:** Keywords with total link strengths in Granger causality.

Keywords	Occurrences	Total Link Strength
Granger causality	3963	12,891
Cointegration	2128	11,166
Economic growth	1590	8321
Time series	1450	7579
CO_2_ emissions	883	6630
Unit root	847	5160
Financial development	725	5098
Energy consumption	616	4326
GDP	449	2986
Income	446	2965

**Table 10:** Citation metrics of Granger causality publications.

Items	Metrics
Extraction Date	13 January 2023
Number of Publications	11,701
Total Number of Citations	274,321
Number of Years	42 (1981–2023)
Citations per Year	6531.45
Citations per Paper	23.44
Citations per Author	118,838.8
Papers per Author	5295.57
Authors per Paper	3.11
*h*-index	209
*g*-index	330

## Data Availability

The data presented in this study are available on request from the corresponding author.
